# An 11-Year Comprehensive Retrospective Study on the Prevalence and Genotype Distribution of Human Papillomavirus (HPV) in Women and Men in Türkiye and Northern Cyprus

**DOI:** 10.3390/jcm15197575

**Published:** 2026-09-29

**Authors:** Tutku Taskinoglu, Zafer Yazici, Seda Gozel, Adem Keskin, Melek Bilgin, Tugce Cuneydioglu, Mehmet Hakan Taskin

**Affiliations:** 1 Department of Medical Microbiology, Faculty of Medicine, University of Ufuk, Ankara 06520, Türkiye; tutku.taskinioglu@ufuk.edu.tr; 2 Department of Virology, Faculty of Veterinary Medicine, Ondokuz Mayis University, Samsun 55270, Türkiye; seda.gozel@omu.edu.tr (S.G.);; 3 Department of Medical Biochemistry, Faculty of Medicine, Aydın Adnan Menderes University, Aydin 09010, Türkiye; adem.keskin@adu.edu.tr; 4 Department of Medical Microbiology, Faculty of Medicine, Samsun University, Samsun 55080, Türkiye; melek.bilgin@samsun.edu.tr

**Keywords:** human papillomavirus, high risk, RT-PCR, genotyping, prevalence

## Abstract

**Background/Objectives**: Human papillomavirus (HPV) is one of the most widespread sexually transmitted infections (STIs) worldwide. The aim of the present study was to evaluate the prevalence and genotype distribution of HPV in Türkiye and Northern Cyprus. **Methods**: In the present study, we analysed HPV results recruited from 37 health centres in Türkiye and Northern Cyprus between 2015 and 2025. Briefly, the presence and genotypes of HPV were detected with two different diagnostic kits. **Results**: A total of 37,458 molecular test results were evaluated in this study. Overall, 9732 (25.98%) individuals who underwent at least one HPV test were positive. The mean age of HPV-positive individuals was 34.87 ± 9.62 years. HPV positivity was higher in women than in men. The highest HPV positivity rate (41.13%) was observed among individuals younger than 25 years. High-risk (HR) HPV accounted for 42.27% of the 9732 HPV-positive individuals identified in this study. The most common HPV genotypes were 16, 18, and 6, respectively. HPV genotypes 42 and 35 were observed more frequently in women than in men, whereas genotypes 39, 59, and 68 were more prevalent in men. HPV 6 and 11 were most observed in individuals younger than 25 years. Multiple-type infections were most frequently observed in individuals younger than 25 years. Single-type HPV infections were commonly detected in those aged 50–59 years and in individuals over 60 years. **Conclusions**: These findings contribute to our understanding of epidemiological data on HPV genotype distribution in Türkiye and provide meaningful comparisons across age groups. This study may increase public awareness of HPV infection and help public health authorities develop effective prevention strategies.

## 1. Introduction

Cervical cancer (CC) is a growing health concern for women worldwide, as it remains the fourth most prevalent malignancy and one of the leading causes of cancer-related mortality. A recent study reported that 604,000 women were diagnosed with CC resulting in 280,000 deaths in 2024 [1]. Human papillomavirus (HPV), recognised as the primary cause of CC, is the most widespread sexually transmitted viral infection across the world and affects not only women but also men [2]. Several factors play a vital role in influencing the clinical and epidemiological profile of HPV-related disease, e.g., age, sex, route of transmission, HIV infection, other forms of immunosuppression, concurrent genital dermatoses, lack of HPV vaccination and sexual habits. Being infected with HR-HPV is not the only factor that induces the persistence of HPV infection and subsequent development of intraepithelial neoplasia and invasive carcinoma [3]. HPV, a non-enveloped virus containing a double-stranded DNA genome, is a member of the *Alphapapillomavirus* genus of the first *Papillomavirinae* subfamily in the *Papillomaviridae family*. Thus far, previous studies have identified more than 200 distinct HPV genotypes [4]. HPV genotypes are categorised into low-risk genotypes (LR-HPV) and high-risk genotypes (HR-HPV). The most common HR-HPV genotypes worldwide are HPV 16, 18, 58, 52, and 33 [5]. In Türkiye, HPV 16, HPV 6, and HPV 11 were reported among the most prevalent genotypes once all HPV genotypes, including both high-risk and low-risk types, were considered [6]. Furthermore, HPV 18, HPV 31, HPV 51, HPV 52, and HPV 56 were reported among other frequently detected genotypes [6,7]. The existing body of research on HPV indicates that persistent infection with HR-HPV is the main etiological factor in the development of CC [5]. While approximately 99.7% of CC diagnoses are associated with HR-HPV subtypes, LR-HPV subtypes (e.g., 6, 11) are generally linked to benign proliferative lesions, including genital warts and recurrent respiratory papillomatosis.

Up to now, there has been limited research directly investigating HPV prevalence in men in contrast to women. What is known about HPV prevalence is largely based on data from studies including high-risk populations (e.g., men who have sex with men and HIV-positive men), as well as those exhibiting symptoms of sexually transmitted infections (STIs). In addition, the high-risk population also includes male partners of women diagnosed with HPV infection or abnormal cervical cytology [8,9].

The aim of this present study was to better understand the characteristics of HPV among both women and men and to assess its prevalence and genotype distribution of HPV in Türkiye and Northern Cyprus. Since HPV is capable of infecting both sexes, this study is important and will also contribute to the field of HPV epidemiology in Türkiye and Northern Cyprus.

## 2. Materials and Methods

### 2.1. Study Population and Specimen Collection

This retrospective study was conducted in Türkiye and Northern Cyprus. Geographically, Türkiye has seven regions: the Marmara, Aegean, Mediterranean, Black Sea, Central Anatolia, Eastern Anatolia, and Southeastern Anatolia regions. HPV molecular test results were retrospectively obtained from laboratory records of 37 healthcare centres located across the study regions, consisting of 17 secondary- and tertiary-care hospitals, 13 polyclinics, and seven private laboratories. The analysed data were retrieved from the records of the respective laboratories at participating centres. HPV positivity and genotype information were obtained from real-time polymerase chain reaction (qPCR) results generated from cervical and vaginal swab specimens and biopsy samples collected from patients with suspected HPV infection. A total of 37,458 HPV test results obtained between January 2015 and December 2025 were included in the analysis after excluding repeat tests. Furthermore, the collected data were also evaluated by age, gender, year of testing, season, province and geographical region to investigate temporal and geographical patterns in HPV positivity and genotype distribution.

### 2.2. Ethical Approval

This retrospective study was conducted following approval from the Non-Interventional Clinical Research Ethics Board of Samsun University (decision number: GOKAEK 2026/1/2; approval date: 1 April 2026). All procedures were conducted in accordance with the principles of the Declaration of Helsinki.

### 2.3. HPV Diagnosis and Genotyping

A real-time RT-PCR method was used for the diagnosis and genotyping of HPV with two different HPV detection kits. (*i*) In total, 77.68% (n = 29,097) of all specimens were tested using a COBAS 4800 HPV kit on a COBAS 4800 instrument (Roche Diagnostic, Manheim, Germany). This method produced results for HPV 16, HPV 18, and other high-risk HPV subtypes (OHR-HPV). (*ii*) A total of 22.32% (n = 8361) of all specimens were tested using a Bosphore HPV HR-LR genotyping kit v4 (Anatolia Geneworks, Istanbul, Türkiye), which can distinguish high-risk (HR) subtypes 16, 18, 26, 31, 33, 35, 39, 45, 51, 52, 53, 56, 58, 59, 66, 68, 69, and 82 and low-risk (LR) subtypes 6, 11, and 42, according to the manufacturer’s protocol.

### 2.4. Statistical Analysis

Statistical analyses were carried out using SPSS version 22 for Windows (IBM, New York, NY, USA). Continuous variables were presented as mean ± standard deviation (mean ± SD), while categorical variables were expressed as n (%). Independent sample *t*-tests and the chi-square tests were used to conduct intergroup comparisons for continuous and categorical variables, respectively. To investigate age-related variation, a quadratic logistic regression analysis was conducted as the primary statistical method, considering age as a continuous variable. In addition, complementary analyses, including linear association tests, were used to evaluate linear trends between age groups (considered as categorical variables) and HPV positivity. Gamma tests were performed to assess the direction of the association. A *p*-value < 0.05 was regarded as statistically significant.

## 3. Results

### 3.1. Study Population and HPV Prevalence According to Gender and Age

The present study included 37,458 samples in total from individuals of both sexes tested for HPV infection. Detailed data distribution of the specimens by gender, age, specimen genotypes and HPV test kits used is given in Table 1. The study population comprised 34,517 women (92.15%) with a mean age of 37.36 ± 9.81 years and 2941 men (7.85%) with a mean age of 35.56 ± 10.04 years. The overall mean age of the study population was 37.22 ± 9.84 years. Women were significantly older than men (*p* < 0.001). Futhermore, the number of women whose test results were evaluated in this study was significantly higher than the number of men. This difference may be largely explained by the more frequent and routinely implemented genital screening practices among women, whereas standardised routine genital screening programs for men are not generally available. The HPV positivity rate was higher in women (26.30%) than men (22.20%) (*p* < 0.001). The mean ages were calculated as 34.87 ± 9.62 years for HPV-positive patients, and 38.05 ± 9.78 years for HPV-negative patients. The mean age of HPV-positive patients was statistically younger than that of HPV-negative patients (*p* < 0.001). A comparison of the age distribution showed that the HPV-positive rate was statistically significant. An investigation into the HPV positivity rate among age groups revealed that the highest positivity rate was detected in those younger than 25 years (41.13%). The first aspect obtained from this analysis revealed that the HPV positivity rate was reduced progressively with rising age, reaching its lowest levels in participants over 50 years old. This shows the rarity of HPV positivity estimated at approximately 56.2 years of age. The linear-by-linear association test demonstrated a statistically significant trend in HPV positivity across age groups (χ^2^ = 747.23, *p* < 0.001). Gamma analysis indicated a weak-to-moderate negative association between age and HPV positivity (γ = −0.206, *p* < 0.001), suggesting that HPV positivity tended to decrease with increasing age. This finding pointed out that HPV positivity decreased with increasing age. The test samples in the study consisted of cervical swabs (88.95%, n = 33,320), genital swabs (7.20%, n = 2698) and biopsy samples (3.83%, n = 1440). A total of 37,458 HPV test results were subsequently obtained. The highest positivity rate was observed from biopsy samples *(p* < 0.001). In total, 25.98% (n = 9732) of the specimens were found to be HPV-positive using a real-time RT-PCR with two different HPV detection kits. When compared, the Bosphore HPV HR-LR kit V4 (Anatolia Geneworks, Türkiye) detected a higher HPV positivity rate than the COBAS 4800 HPV kit (*p* < 0.001). A quadratic logistic regression analysis also indicated a U-shaped relationship between age and HPV positivity.

### 3.2. The Assessment of HPV Positivity by Years and Seasons

In the present study, the HPV positivity rates were evaluated in terms of years and seasons, as detailed in Table 2. The obtained results demonstrated that the highest and lowest rates of HPV positivity were in 2023 and 2017, respectively (*p* < 0.001). A seasonal analysis over an 11-year period revealed that the highest HPV-positive rates were during the summer season, whereas the lowest were during the winter season (*p* = 0.033).

### 3.3. The Geographical Distribution of HPV in Türkiye and Northern Cyprus

As detailed in Table 3, the geographical distribution of HPV was evaluated in 37 centres located in the seven distinct regions of Türkiye and Northern Cyprus (Table 3). The highest HPV positivity was determined in Northern Cyprus, with a rate of 36.81%, while the lowest HPV positivity was in Ankara, with a rate of 22.74% (*p* < 0.01). Regarding the distribution of HPV positivity according to geographic region in Türkiye, the highest and lowest HPV positivity rates were observed in Southeastern Anatolia and Central Anatolia, respectively (*p* < 0.001). Of the 9732 HPV-positive specimens, the genotyping of 5619 (57.73) specimens was carried out, whereas 4113 (42.27%) were evaluated as “other high-risk HPV” (OHR-HPV) without genotype identification. In this context, 31 different HPV genotypes—a total number of 7092—were determined in 5618 specimens.

### 3.4. The Evaluation of HPV Genotypes by Risk Category

As detailed in Table 4, the 7092 genotypes were categorised into three groups: 5351 (75.45%) were classified as high risk (HR), 454 (6.40%) as probably high risk (pHR), and 1287 (18.14%) as low risk (LR). A total of 6459 (91.07%) of 7092 genotypes were also detected in women, whereas 633 (8.93%) were in men. Furthermore, the distributions of HR, LR, and pHR in 6459 HPV-positive women were 4875 (91.08%), 1188 (16.75%), and 396 (5.58%), respectively. Among HPV-positive men, 476 (6.71%), 100 (1.41%), and 57 (0.80%) were classified into the respective risk groups.

### 3.5. The Distribution of Identified HPV Genotypes According to Risk Category and Gender

In line with the obtained results, 16 (HR), 18 (HR), 6 (LR), 52 (HR), 42 (LR), 66 (pHR) and 11 (LR) were most prevalent among HPV genotypes with rates of 40.68%, 12.76%, 9.74%, 5.56%, 3.82%, 3.16% and 3.13%, respectively. HPV 42 and 35 were more frequently observed in women than in men (*p* = 0.046 and *p* = 0.02, respectively). However, genotypes 39, 59, and 68 were more prevalent in men (*p* = 0.029, *p* = 0.006 and *p* < 0.001, respectively) (Table 5).

### 3.6. Single and Mixed HPV Infection

In this study, genotyping was performed on 5618 samples. Of these, 4299 (76.52%) harboured a single HPV genotype, whereas 1224 (21.79%), 35 (0.63%), and 60 (1.07%) samples had dual, triple, and quadruple genotype infections, respectively. Overall, 7092 HPV genotype detections were identified, comprising 4299 (60.62%) single, 2448 (34.52%) dual, 105 (1.48%) triple, and 240 (3.38%) quadruple genotype detections (Table 6).

## 4. Discussion

The aim of our study was to evaluate HPV prevalence by analysing 37,458 results molecularly obtained from 37 centres in Türkiye and Northern Cyprus. The total analysed sample consisted of 92.15% women and 7.85% men. The mean age among female individuals was higher compared to male individuals. In the present study, the overall HPV rate was found to be 25.98%. Previous studies in Türkiye revealed a wide range of HPV positvity rates ranging from 2.1% to 47.7%, depending on age and region [10,11]. Taskin et al. [12] reported a 9.1% prevalence of HPV, whereas Alacam et al. [10] reported it to be 36.3%. The overall HPV prevalence obtained from our study was within this range. In the present study, the overall HPV prevalence among the relevant years was found to be higher in women (26.30%) than in men (22.20%). However, previous studies conducted in other countries indicated that the overall HPV prevalence in men was often found to be equal to or higher than in women [13,14]. Lewis et al. [13] reported that the HPV rate in the USA was 41.8% in men, which was higher than the 38.4% determined in women. Similarly, in a study conducted in China, Ke et al. [14] also reported that HPV prevalence was higher among men than among women, at 41.3% and 22.85%, respectively [15]. The most important reason as to why we found a higher prevalence of HPV in women compared to men in our study was, in our opinion, that HPV prevalence studies conducted to date in Türkiye and other conservative countries have primarily focused on women, and studies concerning HPV have been limited in men. Consistent with our findings, in a study conducted in Iran, Mojarrada et al. [16] reported an overall HPV prevalence of 43% among 447 samples. In the same study, 12 of the 16 male patients tested were HPV-positive, corresponding to a positivity rate of 75%, whereas 183 of the 431 samples collected from women were HPV positive, corresponding to a positivity rate of 42.5%. However, when calculated based on the total number of samples, the HPV positivity rates among women and men were 40.93% and 2.68%, respectively, indicating a substantially higher proportion of HPV-positive samples in women than in men [16]. Furthermore, the observed sex-based difference in our study can be explained by the predominance of women participants in the sample size. Collectively, these factors may explain the higher HPV positivity rate detected among women within this study.

In the present study, the obtained results indicated that there was a decrease in the overall HPV positivity rate depending on increasing age. This age-dependent decrease may reflect the natural course of HPV infection since most HPV infections are transient and gradually eliminated by the immune system response over time. Whereas HPV infection acquisition occurs most frequently among younger age groups, the rate of new infections is reduced in older age groups due to diminished exposure and reduced rates of new sexual partners. A study performed in China with 78,355 women participants showed higher HPV infection rates in those under 25 years and over 50 years [17]. Another study conducted in China revealed a bimodal age distribution of HPV prevalence across age groups, characterised by peaks detected in females under 21 years as well as older than 61 years [18]. A study from Türkiye reported that HR-HPV positivity reached its highest level among those aged 21–24 years (31.97%) [19]. In the current study, the peak HPV positivity rate was detected in individuals under 25 years, in line with prior findings. Within younger age groups, HPV infections are mainly newly acquired and transient, representing a stage in which effective genotype-specific immune responses are not yet completely developed [20]. In the follow-up study by Arthur et al. [21], 36 HPV genotypes were evaluated in 502 female university students followed for 24 months. During this period, 40.4% of female students were infected with a new HPV infection, while differences in clearance rates were determined across HPV genotypes. These findings indicated that the continuous acquisition and clearance of different HPV genotypes among younger individuals reflected the dynamic nature of HPV infection. In young age groups, behavioural factors, including a higher number of sexual partners and inconsistent condom use, may increase the risk of HPV acquisition and transmission, whereas consistent condom use is one of the important preventative measures against HPV infection and may reduce risk [22,23]. In addition, suboptimal HPV vaccination coverage or delayed initiation of vaccination may be associated with the higher rate of HPV prevalence reported in younger age groups [24,25,26]. As a result, a smaller percentage of long-term infection can still be detected at older ages. In women, age-related hormonal and anatomical changes of the cervix (e.g., regression of the transformation zone after menopause) may additionally decrease HPV detection rates [27]. Furthermore, differences in screening practices and risk characteristics in older populations may play a role in the decrease in HPV positivity rates. These results are consistent with the recognised natural history of HPV infection because HPV acquisition generally occurs early, following sexual activity.

When the distribution of cases by year and season was evaluated, the highest number of HPV-positive cases was observed in 2023 (30.16%), particularly during the summer months. Study samples were collected from 37 centres in seven different geographical regions, as well as Northern Cyprus. Among the regions examined, Northern Cyprus presented the highest rate of HPV positivity (36.81%). A retrospective study over 11 years carried out in Northern Cyprus documented a total HR-HPV prevalence of 44.3% in all individuals [28]. In another study in Cyprus in 2017, the overall HPV prevalence was reported to be 72.8%, and the HR-HPV rate was 34.8%. Our study and previous studies considered that HPV positivity was high in Cyprus [29]. The results of this present study may be attributed to various local factors such as increased population mobility linked to tourism, international students, and a large transient population. These factors may promote HPV transmission through more extensive sexual networks. Moreover, the comparatively young population structure of Northern Cyprus may be associated with higher rates of HPV infection since HPV acquisition is most frequently observed among younger individuals.

In Türkiye, it was illustrated that the highest rate of HPV positivity was detected in the Southeastern Anatolia region. This result may be explained by a combination of social, financial, cultural, and healthcare-related factors [30]. Collectively, the primary factors contributing to increased rates of undiagnosed and persistent HPV infections may include the limited availability of HPV vaccines, lower immunisation rates, and lower participation in organised cervical cancer screening programmes [31]. Furthermore, early marriage age, higher parity, and longer sexual exposure period may increase the probability of HPV infection and its persistence. Limited awareness of sexual health and inconsistent condom use are likely to increase the HPV transmission. Additionally, it cannot be eliminated that there is a selection bias, meaning that individuals tested for HPV may represent a high-risk subgroup. Collectively, these factors likely account for the higher HPV positivity rates reported in Southeastern Anatolia and indicate the importance of region-specific public health interventions promoting vaccination, screening, and education [32]. However, it was reported that the prevalence of HPV in southeastern Türkiye is lower than the global average. This study also found that HR-HPV genotypes were the most frequently observed in this area, and HPV prevalence was highest at older ages relative to other regions worldwide [30]. Among HPV-positive individuals, 42.27% (4113/9732) were determined to have OHR-HPV. A total of 93.29% (9079/9732) of HPV positivity rates were mainly observed among women, and a significantly lower proportion was observed among men (6.71%).

In this study, the most prevalent HPV genotypes were 16, 18, and 6, respectively. In comparison, a systemic review and meta-analysis indicated that the most frequently observed HPV genotypes related to cervical intraepithelial neoplasia (CIN) in China were HPV 16, 52, and 58 [33]. Similarly, the most frequently observed HR-HPV genotypes among Thai women included HPV 52, HPV 16, and HPV 51 [22]. A study conducted in Türkiye reported that HPV 16 was the most common high-risk HPV genotype in cases of atypical squamous cells of undetermined significance and low-grade squamous intraepithelial lesions, whereas HPV 18 was the most frequently identified genotype in high-grade squamous intraepithelial lesions [34]. In our previous study regarding HPV prevalence in Samsun Province, the most detected HPV genotypes were 16 and 18 [12]. Our findings are consistent with those reported in numerous previous studies. In the present study, the most common genotypes were found to be 16, 18 and 6, respectively, once HPV genotypes were classified according to both sexes. Based on cervical smear results from the Central Anatolian region, Fındık et al. [6] identified the following HPV-positive genotypes in women, listed in decreasing order of frequency: HPV 16; HPV 16 with multiple infection; HPV 31; HPV 51; HPV 39; HPV 52; HPV 56; HPV 18; HPV 68; HPV 35; HPV 18 with multiple infection; HPV 58; HPV 45; HPV 59; HPV 16; HPV 18 with multiple infection; HPV 16; HPV 18; and HPV 33. A study conducted among university students in Gaborone, Botswana, reported that the five most prevalent HR-HPV genotypes in women were HPV 51, HPV 39, HPV 66, HPV 35, and HPV 59, whereas the most common genotypes in men were HPV 66, HPV 51, HPV 35, HPV 59, and HPV 58 [35]. Ke et al. [14] found that the most common HR-HPV genotypes among women were HPV 52, HPV 58, and HPV 16, whereas in men, the most common genotypes were HPV 52, HPV 16, and HPV 51. Among the HR-HPV genotypes detected in our study population, HPV 16 was the most prevalent genotype among men, accounting for 39.49% of cases, followed by HPV 18, HPV 59, HPV 39 and HPV 51, respectively. Ma et al. [36] revealed that HPV genotypes 16, 52, 51, 18, 39, and 59 were the most commonly observed HR-HPV genotypes among men in Northern China. Bruni et al. [9] reported that the most commonly observed HPV genotypes in men were 16, 52, 51, 39, and 18, respectively. The results obtained in men in the present study were consistent with these findings. Furthermore, Kusters et al. [37] found no significant difference in HPV prevalence between men who have sex with men (MSM) and men who have sex with women (MSW) and identified HPV 6 (LR) and HPV 16 (HR) as the most common genotypes in both groups. In another study, the prevalence of HPV and HR-HPV in MSM was reported to be higher than in MSW [38]. Our study did not examine clinical details relating to HIV-positive individuals, MSM or other pre-defined high-risk male populations. However, Marra et al. [39] reported that HPV 16 prevalence was higher in HIV-positive MSM than in HIV-negative MSM (30% vs. 14%) and was also higher in HIV-positive MSW than in their HIV-negative counterparts (11% vs. 3%). These findings indicate that both sexual practices and HIV status may influence type-specific HPV prevalence in men. Another systematic review and meta-analysis of genital HPV infection in men reported a pooled prevalence of 31% for any HPV and 21% for high-risk HPV, with HPV 16 being the most frequently detected genotype [40]. In our study (22.20%), overall HPV positivity in men was within the range reported in the international literature, although direct comparisons remain limited by differences in population characteristics and sampling strategies. The prevalence of HPV genotypes shows variation among different countries and is affected by the number of sexual partners, condom use, male circumcision prevalence, HPV awareness, and screening and vaccination strategies [41]. In our study, the correlation between HPV genotypes and age categories was also evaluated, and HPV genotypes 16 and 18 were found to be the most prevalent in individuals aged between 30 and 39 years (Table 2). In a previous study conducted in the city of Samsun, HPV genotype 16 was most frequently detected between ages 30 and 39 [12]. HR-HPV 16 and 18 were associated with a greater potential for persistent infection, and persistent HPV infections could be detected more frequently in adult age groups [42,43]. However, the cross-sectional design of the present study does not allow us to distinguish persistent infections from recently acquired infections, and this interpretation should therefore be considered a possible explanation rather than a demonstrated mechanism. Several studies on HPV have demonstrated that HR-HPV 16 prevalence may decline after early adulthood but may display a secondary increase in older age groups (e.g., those in their 50s), possibly because of the reactivation of latent infection or age-related changes in immune responses [44]. In their study, Argyri et al. [45] found that HPV 16 was most common in the 20–24 age group, with the second highest prevalence observed in the 50–54 age group. In a large population-based study of women attending primary HPV-based cervical screening, the HPV 18 prevalence was most frequently detected among women aged 30–35 years (a group representing younger eligible screening participants) and was generally lower in older cohorts. This indicates age-based differences in HPV 18 infection patterns [46]. In contrast, HPV genotypes 6 and 11 were predominantly observed in those younger than 25 years. This pattern may be explained by differences in transmission dynamics, clinical behaviour, and interactions with the immune system. HPV genotypes 6 and 11 are commonly transmitted soon after the initiation of sexual activity, and those aged below 25 years represent the population with the greatest incidence of first HPV exposure. Consequently, newly acquired infections are more frequently detected in this age group. In a very large observational study of over 180,000 women, HPV 6 and HPV 11 infection prevalence showed a U-shaped (bimodal) distribution with age—highest in the <20-year-old group, lower in the middle age bracket (20–49), and with a modest increase again in the older age group (≥60) [47]. The multinational HPV infection in a study on men found that the seroprevalence of HPV genotypes including HPV 6 and HPV 11 increased with age among young-to-middle-aged men, suggesting that cumulative exposure and immune response vary with age and sexual behaviour patterns [48]. Our results are consistent with these studies. We also found that HPV genotype 61 was most prevalent in the 50–59 age group. A physiological decline in cellular immune function is observed after the age of 50, which may impair viral clearance. Low-risk HPV genotypes, such as HPV 61, are generally cleared more efficiently at younger ages; however, viral clearance tends to increase with advancing age, allowing for infections to remain detectable for longer periods in older individuals. Li et al. [49] found that HPV genotype 61 was common among individuals aged ≥ 55 years.

In the present study, genotyping was performed on 5618 HPV-positive specimens, from which a total of 7092 genotypes were identified. Of these specimens, 4299 (76.52%) harboured a single genotype, whereas 1224 (21.79%), 35 (0.63%), and 60 (1.07%) harboured dual, triple, and quadruple genotypes, respectively. Among the 7092 genotypes identified, 4299 (60.62%) were single genotypes. Especially, the most frequent single-type infections were 16, 18, 6, and 11, which harboured HPV genotypes covered by the bivalent vaccine (HPV 16 and 18) as well as the quadrivalent vaccine (16, 18, 6, and 11), suggesting that a considerable proportion of infections in this cohort might theoretically have been avoided through vaccination. Furthermore, dual, triple, and quadruple genotypes were detected among HPV-positive samples in 2448 (34.52%), 105 (1.48%), and 240 (3.38%) cases, respectively. The present findings imply that the usage of bivalent, quadrivalent and nonavalent vaccines may lead to a greater reduction in HPV-related infections across the population. The high rate of co-infection with HPV 6 and HPV 11 may be due to their common transmission pathways, similar biological characteristics, and strong association with genital warts. Their strong phylogenetic similarity and capacity to coexist without marked immune-related competition may further enhance simultaneous infection. Consistent with this observation, a study conducted in Australia revealed that HPV was observed in 90.8% of genital wart cases, with HPV 6 and 11 responsible for 86% of infections. The same study also mentioned that HR-HPV genotypes were detected in 31% of cases [50]. Similarly, a study carried out in Hong Kong among male patients with genital warts indicated that HPV 6 and/or HPV 11 alone accounted for 63.1% of lesions [51].

Finally, the results of our study demonstrated that the high frequency of HPV infection in those younger than 25 years aligns with greater exposure and the transient nature of HPV infections in this age group. This pattern may be explained by behavioural risk factors such as early sexual life, a higher number of sexual partners and a high risk of simultaneous exposure to several HPV genotypes. Additionally, many infections remain transient and have not yet been eliminated by the immune system. It is noteworthy that HPV infections were more often identified among those aged 50–59 and ≥60 years. This observation may suggest the elimination of transient infections acquired earlier in life, with only persistent infections remaining detectable over time. Furthermore, age-associated alterations in immune function and the possible reactivation of latent infections may play a vital role in this observed pattern.

In the present study, there were several limitations that should be considered when interpreting the results. (*i*) The ability to establish temporal or causal relationships between HPV infection and potential risk factors could be limited due to the retrospective and laboratory-based study. (*ii*) The selection of the study population did not rely on population-based random sampling. For this reason, selection bias cannot be underestimated. (*iii*) The comparability of HPV prevalence between female and male patients may have been limited by the unequal numbers of patients of each sex, which may have contributed to the lower observed HPV prevalence among men. (*iv*). The knowledge gap regarding the important demographic and clinical risk factors, including the number of sexual partners, age at sexual debut, condom use, circumcision status, immunosuppression, and HPV vaccination status, was also among the limitations. (*v*) An additional limitation of this study was the use of two different HPV kits. As these tests differ in terms of features, the detection and reporting of specific HPV genotypes may not be directly comparable across all study years and participating centres.

## 5. Conclusions

A comprehensive evaluation of HPV positivity and genotype distribution in Türkiye and Northern Cyprus was provided in this retrospective study. The overall HPV positivity rate was found to be higher in women than in men. The findings of our study showed that HPV positivity overall followed an age-dependent pattern, with the highest prevalence observed in individuals younger than 25 years, and a progressive decline was also determined as age increased. Furthermore, there was a substantial geographic variation in this study. Northern Cyprus had the highest HPV positivity rate, while among the regions of Türkiye, Southeastern Anatolia showed the highest positivity rate. HPV 16, 18 and 6 were the most frequently detected genotypes. HPV genotype distribution varied by age, with HPV 16 and HPV 18 more frequently detected in younger individuals, while HPV 6 and HPV 11 were predominantly detected in individuals younger than 25 years. HPV 61 was most frequently observed in the 50–59-year-old age group. Among genotyped specimens, single-type infections predominated according to triple and quadruple infections. The frequent detection of HPV 16, 18, 6 and 11 showed that a substantial proportion of HPV infections involved genotypes targeted by currently available HPV vaccines. As a result, these findings indicated considerable variation in HPV positivity according to sex, age, geographic region, and genotype and presented epidemiological data that may contribute to the interpretation of HPV distribution in Türkiye and Northern Cyprus. Given the retrospective, non-population-based study design and the limited availability of behavioural and clinical data at the individual level, it is recommended that the associations observed in this study should be interpreted with caution and validated in future population-based longitudinal studies.

## Figures and Tables

**Table 1:** The distribution of the specimens by gender, age, specimen types and HPV test kits used.

Features of Specimens	Tested (n)	Positive n (%)	*p*-
			Value
*Gender*			
Women	34,517	9079 (26.30)	<0.001
Men	2941	653 (22.20)	
*Age Groups*			
<25 years old	2873	1182 (41.13)	<0.001
25–29	6195	2138 (34.50)	
30–39	14,158	3608 (25.48)	
40–49	10,109	2026 (20.04)	
50–59	3197	611 (19.11)	
60 and over	926	168 (18.18)	
*Specimens*			
Cervical swab	33,320	8383 (25.16)	<0.001
Genital swab	2698	412 (15.27)	
Biopsy	1440	937 (65.07)	
*Test kits used*			<0.001
Cobas 4800 HPV	29,097	7193 (24.72)	
Anatolian Geneworks HPV	8361	2539 (30.37)	

**Table 2:** The distribution of individuals with HPV according to year and season.

Features		Tested (n)	Positive n (%)	*p* Value
*Years*				
	2015	2973	821 (27.62)	<0.001
	2016	4416	948 (21.47)	
	2017	2986	427 (14.30)	
	2018	2356	664 (28.18)	
	2019	2742	752 (27.43)	
	2020	2426	649 (26.75)	
	2021	3131	920 (29.83)	
	2022	3011	843 (28.00)	
	2023	3309	998 (30.16)	
	2024	4892	1312 (26.82)	
	2025	5216	1398 (26.80)	
*Season*				
	Spring	9127	2368 (25.94)	0.033
	Summer	8710	2356 (27.05)	
	Autumn	9876	2557 (25.89)	
	Winter	9745	2451 (25.15)	

**Table 3:** Distribution of HPV-positive individuals by sample collection centres and geographic regions.

Provinces and Regions Specimen Collected		Tested (n)	Positive n (%)	*p*- Value
*Provinces*		37,458	9732 (25.98)	
	Istanbul	14,896	3806 (25.55)	**<0.001**
	Ankara	10,774	2450 (22.74)	
	Adana	1693	488 (28.82)	
	Mersin	1604	500 (31.17)	
	Izmir	1093	260 (23.79)	
	Antalya	995	325 (32.66)	
	Bursa	926	264 (28.51)	
	Northern Cyprus	883	325 (36.81)	
	Hatay	783	238 (30.40)	
	Bolu	505	159 (31.49)	
	Kırıkkale	481	127 (26.40)	
	Maraş	473	113 (23.89)	
	Denizli	439	113 (25.74)	
	Diyarbakır	302	99 (32.78)	
	Samsun	242	66 (27.27)	
	Balıkesir	219	54 (24.66)	
	Others	1150	345 (30.00)	
*Regions*		37,458	9732(25.98)	
	Mediterranean	6501	2000 (30.76)	**<0.001**
	Central Anatolia	11,464	2632 (22.96)	
	Marmara	16,181	4174 (25.80)	
	Aegean	1689	427 (25.28)	
	Black Sea	900	279 (31.00)	
	Southeastern Anatolia	587	186 (31.69)	
	East Anatolian	136	34 (25.00)	

**Table 4:** The distribution of HPV according to risk category and gender.

Risk Category	Tested n (%)	Gender	
		Women n (%)	Men n (%)
High-risk (HR)	5351 (75.46)	4875 (68.73)	476 (6.71)
Low-risk (LR)	1287 (18.14)	1188 (16.75)	100 (1.41)
Probably High-risk (pHR)	454 (6.40)	396 (5.59)	57 (0.8)
**Total**	**7092 (100)**	**6459 (91.07)**	**633 (8.93)**

**Table 5:** The number and percentages of identified HPV by risk category and gender.

HPV Genotype	Risk Category	Identified n (%)	Gender		*p*-Value
			Women n (%)	Men n(%)	
16	HR	2885 (40.68)	2635 (40.80)	250 (39.49)	0.525
18	HR	905 (12.76)	828 (12.82)	77 (12.16)	0.637
6	LR	691 (9.74)	630 (9.75)	61 (9.64)	0.924
52	HR	394 (5.56)	358 (5.54)	36 (5.69)	0.880
42	LR	271 (3.82)	256 (3.96)	15 (2.37)	**0.046**
66	pHR	224 (3.16)	198 (3.07)	26 (4.11)	0.153
11	LR	222 (3.13)	202 (3.13)	20 (3.16)	0.965
51	HR	212 (2.99)	196 (3.03)	16 (2.53)	0.475
59	HR	160 (2.26)	136 (2.11)	24 (3.79)	**0.006**
45	HR	144 (2.03)	134 (2.07)	10 (1.58)	0.400
39	HR	133 (1.88)	114 (1.76)	19 (3.00)	**0.029**
35	HR	126 (1.78)	122 (1.89)	4 (0.63)	**0.022**
56	HR	119 (1.68)	109 (1.69)	10 (1.58)	0.840
58	HR	108 (1.52)	96 (1.49)	12 (1.90)	0.422
68	pHR	101 (1.42)	75 (1.16)	26 (4.11)	**<0.001**
31	HR	99 (1.40)	90 (1.39)	9 (1.42)	0.954
70	pHR	96 (1.35)	92 (1.42)	4 (0.63)	0.100
61	LR	89 (1.25)	85 (1.32)	4 (0.63)	0.140
33	HR	54 (0.76)	47 (0.73)	7 (1.11)	0.331
53	pHR	29 (0.41)	28 (0.43)	1 (0.16)	0.511
73	HR	12 (0.17)	10 (0.15)	2 (0.32)	0.291
55	LR	6 (0.08)	6 (0.09)	0 (0.00)	-
*54*	*LR*	*3 (0.04)*	*3 (0.05)*	*0 (0.00)*	-
65	LR	2 (0.03)	2 (0.03)	0 (0.00)	-
15	LR	1 (0.01)	1 (0.02)	0 (0.00)	-
21	LR	1 (0.01)	1 (0.02)	0 (0.00)	-
26	pHR	1 (0.01)	1 (0.02)	0 (0.00)	-
40	LR	1 (0.01)	1 (0.02)	0 (0.00)	-
62	LR	1 (0.01)	1 (0.02)	0 (0.00)	-
69	pHR	1 (0.01)	1 (0.02)	0 (0.00)	-
82	pHR	1 (0.01)	1 (0.02)	0 (0.00)	-
**Total**		**7092**	**6459**	**633**	

**Table 6:** Prevalence of single and mixed HPV infection according to positive-case genotyping made (no. 5618) as well as the number of identified genotypes from 5618 specimens (no. 7092).

Genotype	Frequency n (%)	Identified n (%)
single	4299 (76.52)	4299 (60.62)
dual	1224 (21.79)	2448 (34.52)
triple	35 (0.63)	105 (1.48)
quadruple	60 (1.07)	240 (3.38)
**Total**	**5618**	**7092**

## Data Availability

The original contributions presented in this study are included in the article. Further inquiries can be directed to the corresponding authors.
